# Eliciting Callus Cultures for the Production of Cytotoxic Polyphenolics from *Maesa indica* Roxb. Sweet

**DOI:** 10.3390/plants13141979

**Published:** 2024-07-19

**Authors:** Fatma Alzahra M. Abdelgawad, Seham S. El-Hawary, Essam M. Abd El-Kader, Saad Ali Alshehri, Mohamed Abdelaaty Rabeh, Ahmed Fathi Essa, Aliaa E. M. K. El-Mosallamy, Rania A. El Gedaily

**Affiliations:** 1 Department of Pharmacognosy, Faculty of Pharmacy, Heliopolis University, Cairo 11785, Egypt; fatma.mahrous@hu.edu.eg; 2 Department of Pharmacognosy, Faculty of Pharmacy, Cairo University, Giza 11562, Egypt; seham.elhawary@yahoo.com; 3 Department of Timber Trees Research, Horticultural Research Institute (ARC), Giza 12619, Egypt; eltorifi_ola@yahoo.com; 4 Department of Pharmacognosy, College of Pharmacy, King Khalid University, Abha 62251, Saudi Arabia; salshhri@kku.edu.sa (S.A.A.); mrabeh@kku.edu.sa (M.A.R.); 5 Department of Natural Compounds Chemistry, National Research Center, 33 El Bohouth Street, Cairo 12622, Egypt; ahmedfathyessa551@gmail.com; 6 Department of Pharmacology, Medical Division, National Research Centre, 33 El Bohouth Street, Cairo 12622, Egypt; aliaamoneer@hotmail.com

**Keywords:** *Maesa indica*, callus, tissue culture, elicitation, LC-ESI-MS/MS-MRM, cytotoxicity, molecular docking, cancer

## Abstract

*Maesa indica* Roxb. Sweet is a shrub known for its richness in secondary metabolites. A callus culture protocol was established to enhance its chemical profile. Sixteen elicitation culture treatments were evaluated, and we confirmed that the treatment of 200 mg/L polyethylene glycol (4000) coupled with exposure to 30 W UV irradiation for 60 min (PEG4) resulted in the highest total phenolic and total flavonoid contents, which were 4.1 and 4.9 times those of the plant ethanolic extract and 4.9 and 4.8 times those of a control sample, respectively. The phenolic compounds in the different treatments were identified qualitatively and quantitatively using the LC-ESI-MS/MS-MRM technique. Molecular docking studies of the phenolic compounds were conducted using MOE software and revealed that rutin showed the highest binding affinity toward the anti-cancer target (p38*α* MAPK). The cytotoxicity of the ME and PEG 4 treatment was tested against colon, breast, prostate, lung, and liver cell lines using an MTT assay. The highest cytotoxic effect of PEG4 was against prostate cancer with an IC_50_ value of 25.5 µg/mL. Hence, this study showed enhanced secondary metabolite accumulation and identified the phenolic compounds in the 16 treatments. The cytotoxicity assay highlighted the possible cytotoxic effect of the PEG4 treatment, and we recommend further investigations into its activity.

## 1. Introduction

*Maesa indica* Roxb. Sweet is a tall, evergreen shrub indigenous to China and Southern India and often known as wild berry or wild tea [1]. *M. indica* can grow in evergreen, semi-evergreen, and high-humidity forests. *M. indica* has been recently cultivated in Egypt at El-Mazhar Botanical Garden. The plant is rich in several phytochemicals, such as flavonoids, phenolic acids, saponins, tannins, carbohydrates, fixed oil, and glycosides [2]. Several pharmacologically active metabolites have been reported in *M. indica,* including palmitic acid, chrysophanol, 2,3-dihydroxypropyl octadeca-9,12- dienoate kiritiquinon, glyceryl palmitate, stigmasterol, β-sitosterol, dodecane, maesaquinone, quercetin 3-rhaminoside, rutin, chlorogenic acid, catechin, quercetin, nitrendipine, and *β*-thujone [2,3]. This plant is traditionally used in treating cancer, boils, gallstones, lactopenia, paralysis, and puerperalism [2]. The plant is reported to have many biological activities, including antimicrobial, antitumor, antimutagenic, and anthelminthic activities [2]. It also shows strong larvicidal, radical scavenging [4], anti-human coronavirus [5], and antiarthritic activities [6]. A study on *M. indica* concluded that its extract has a strong antidiabetic effect [7]. In addition, it has lung protection activity against potassium-dichromate-induced lung injury [8].

Even though *M. indica* has myriad biological activities, traditionally, the plant has a scare distribution in Egypt and has not produced fruit due to the different environment. Hence, it is of great interest to establish a tissue culture protocol for conserving and enhancing the productivity of such an important plant. To the best of our knowledge, there are no studies of tissue cultures of *M. indica*. The in vitro propagation of four other species of the genus *Maesa*, including *M. argentea*, *M. balansae*, *M. lanceolata,* and *M. perlarius*, has been studied and revealed that multiple shoots are induced through both axillary bud formation and adventitious shoot regeneration from leaf explants [9]. Moreover, a tissue culture study was established from the shoot tips of *M. ramentacea* A. DC. Shoot proliferation was enhanced with Murashige and Skoog (MS) medium enriched with either benzyladenine (BA), BA–kinetin, or BA and naphthalene acetic acid (NAA) combinations. And for rooting, the individual shoots were planted on a root induction medium supplemented with 2 mg/L NAA, and then the plantlets were acclimatized to and transplanted into soil [10]. Another study concluded that *M. perlarius* could be regenerated from leaf-derived callus. Shoots were induced in MS medium in the presence of thidiazuron in combination with *α*-naphthalene acetic acid [11].

In this study, the callus induction strength of plant growth regulators (auxins and cytokines) was investigated on *M. indica* leaf explants. Benzyl amino purine (BAP) has been reported to have a good callus induction capacity for *Trachyspermum ammi* plants, especially when it is combined with 2, 4 dichlorophenoxy acetic acid (2, 4-D) [12]. Picloram is reported as a strong callus induction hormone for various explants (leaf sheaths, leaves, and roots) of *Zingiber officinale var* [13]. Different concentrations and combinations of 2-iso-pentenyl adenine (2 iP) with 2, 4 dichlorophenoxy acetic acid (2, 4-D) were confirmed to have a powerful callus induction ability from hypocotyl, root, and cotyledonary leaf segments of *Withania somnifera* (L.) [14]. The callus induction effect of different media compositions of 2, 4-D dicamba and picloram in wheat (*Triticum aestivum* L.) was reported according to [15]. According to [16], the addition of casine hydrolysate to the medium increased the fresh weight and callus formation within each level of many growth hormones, like 2, 4 dichlorophenoxy acetic acid (2, 4-D), from immature inflorescence explants of a variegated bermudagrass, *Cynodon dactylon* (L.) Pers. cv. “Zebra”.

Moreover, we aimed to enhance the chemical profile of *M. indica* in in vitro cultures by using elicitation techniques, including chemical and physical elicitors, to enhance the production of flavonoid and phenolic compounds. Elicitation can be used for the large-scale production of secondary metabolites in a short period, which could be a suitable solution for increasing the industrial production of secondary metabolites to meet demand [17]. Elicitation promotes chemical defense in plants and activates the biosynthetic pathways of the secondary metabolites, leading to its accumulation [18]. In the present protocol, the elevation of the phenolic compounds’ content was targeted due to their importance. Phenolics are a common class of bioactive metabolites with valuable health-benefiting and disease-preventing characteristics [19]. Additionally, they have strong anti-inflammatory, antioxidant, anticancer, antiviral, antibacterial, anti-hepatotoxic, anti-allergic, and cardioprotective properties [20]. The elicitation study investigated phenolic compound production using H_2_O_2,_ sucrose, sorbitol, polyethylene glycol (4000), and UV-C irradiation, as these have been previously reported to have elicitation power in secondary metabolite production, especially polyphenolic compounds. It has been reported that the use of hydrogen peroxide (H_2_O_2_) as an elicitor caused an elevation in the production of phenolic compounds and enhanced the phytochemical profile of chickpea sprouts [21]. It was found that the addition of sugars (sucrose and sorbitol) as major components to the culture medium induces signaling responses that affect plant development, growth, metabolism, and the production of secondary metabolites [22]. Osmotic stress is a key factor affecting secondary metabolite formation and plant growth. Sugars have been used as a natural osmotic stress material [23]. Moreover, the non-toxic polyethylene glycol 4000 (PEG) induces osmotic stress, which enhances secondary metabolite production [24]. On the other hand, physical elicitation, represented as exposure to UV-C irradiation, has been reported to increase the production of secondary metabolites in cell cultures [25]. So, we assessed the effect of different concentrations of sucrose, sorbitol, and PEG as chemical elicitors, UV-C irradiation as a physical elicitor, and the coupling of physical and chemical elicitation on the production of flavonoids and phenolic compounds in cell cultures. Further, the chemical constituent of each treatment was investigated qualitatively and quantitatively using an LC-ESI-MS/MS in MRM mode. This method of identification is characterized by its high levels of selectivity and sensitivity; therefore, it is the first choice for the analysis of complex plant extracts and can differentiate between many compounds with the same parent ions but differing in fragment ions [26]. The detector was used in MRM (multiple reaction monitoring) screening mode with different standard phenolic compounds.

Recently, natural cytotoxic drugs have attracted researchers’ interest due to their safety and limited adverse effects. *M. indica* was previously reported to have antimutagenic [27] and antitumor [28] activities, in addition to its traditional use in China to treat cancer [29], so we determined it would be interesting to study the anticancer activity of *M. indica* cultivated in Egypt. According to [30], the most prevalent malignancies in Egypt are breast, liver, colorectal, and cervical cancers. Breast cancer is the most common disease among Egyptian women and accounts for 29% of all cases reported to the National Cancer Institute [31].

Furthermore, molecular docking studies of the identified compounds were conducted to predict the role of these compounds in the cytotoxic activity of *M. indica.* Molecular docking is one of the fundamental methods for simulating how a target site may bind based on specific features [32]. Mitogen-activated protein kinases (MAPKs) are an important class of signaling systems that are responsible for mediating cellular responses through the cascade amplification of phosphorylation. Between the subfamilies of MAPKs, p38 MAPK, especially its *α* form, has a vital role in the processes of apoptosis and tumor transformation [33]. Studies have revealed that p38*α* MAPK is closely associated with the development of several cancers, and its inhibition exerts a synergistic effect with other chemotherapeutic agents [34]. So, p38*α* MAPK is considered one of the most attractive anti-cancer targets.

Hence, the current study aimed to establish a callus culture protocol to study the effects of physical and chemical elicitors on the production of flavonoid and phenolic compounds. Moreover, the present study targeted the identification of phenolic compounds in all of the tissue culture treatments qualitatively and quantitatively using LC-ESI-MS/MS in MRM mode as well as conducting molecular docking studies for these identified compounds to explore their binding affinity toward the anticancer target p38*α* MAPK. Ultimately, the cytotoxic activities of the induced callus extract and the mother plant extract were investigated and compared to each other.

## 2. Results and Discussion

### 2.1. Tissue Culture Results

#### 2.1.1. Callus Induction

**Figure 1:**
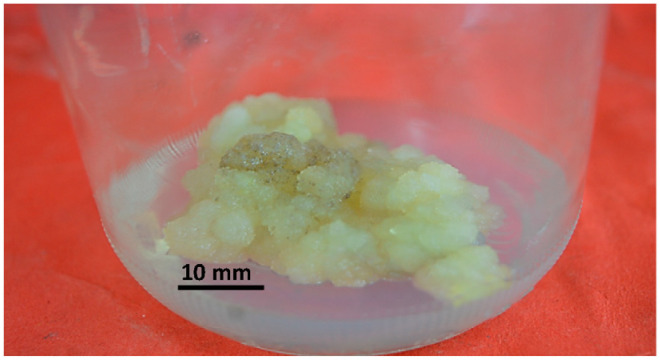
Fully grown callus of *Maesa indica* on MS medium supplemented with 0.25 gm/L CH and 4 mg/L picloram.

Callus induction represents the first stage of secondary metabolite production. The application of plant growth regulators such as auxins and cytokinins at different ratios plays an essential role in callus initiation, growth, and the induction of secondary metabolites [35]. The callus culture protocol for the *Maesa indica* plant was established using its leaves. From all of the 18 treatments prepared for callus initiation, the highest callus mass production, 7.5 ± 0.854 gm, was obtained from treatment No. 16 (the MS medium supplemented with 0.25 gm/L CH and 4 mg/L picloram). The highest level of callogenesis may be due to the callus induction power of picloram and casein hydrolysate. Picloram is considered to be one of the callus induction hormones that enhance callus formation, growth, and secondary metabolite production [13]. CH also enhances callogenesis [36], and its addition to the media in synergy with picloram increases its callus induction power and enhances callus growth, which explains the high weight of the formed callus. The average callus weight of each treatment is represented in Table 1, while the callus grown using treatment No 16. is shown in Figure 1.

#### 2.1.2. In Vitro Secondary Metabolite Production

Sixteen elicitation treatments were used to enhance the chemical profile of the plant, and each contained different chemical and/or physical elicitors with different concentrations. All the tested treatments contain the basal MS media with 25 gm/L of sucrose and 7 gm/L agar, and the treatments that used elicitors with sucrose have extra sucrose; for example, treatment No. contained 50 gm/L sucrose, treatment No. 6 contained 50 gm/L sucrose, treatment No. 7 contained 75 gm/L sucrose, and treatment No. 8 contained 75 gm/L sucrose. For each treatment and the mother plant sample, the measured total phenolics and total flavonoids are illustrated in Table 2.

**Figure 2:**
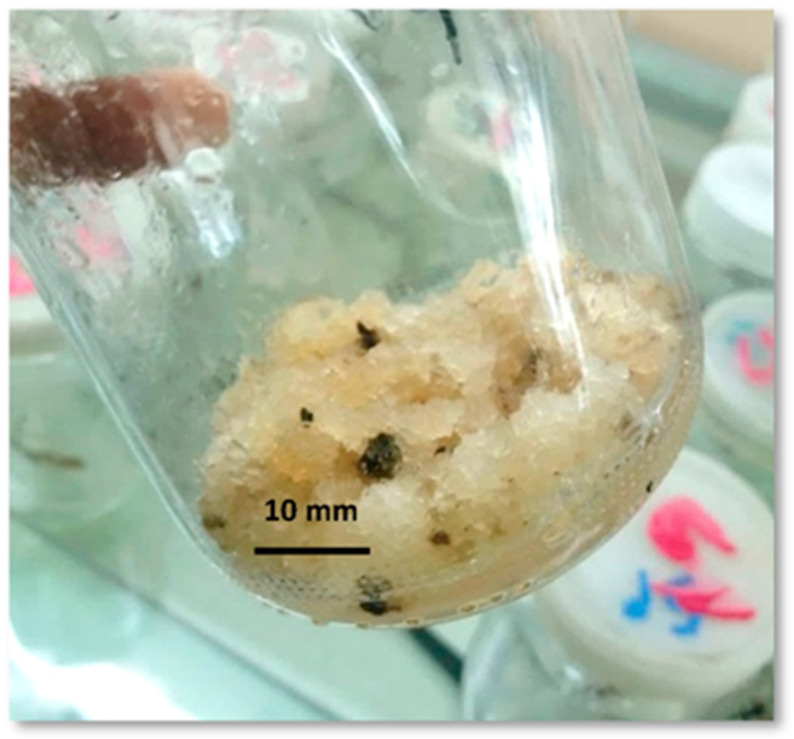
The fully grown callus was produced by treatment No. 16 (200 mg/L polyethylene glycol (4000) + 30 W UV for 60 min.).

The presented data in Table 2 indicate a variation in the total phenolic and total flavonoid contents as a result of the different elicitation effects of each treatment. The highest total phenolic content (48.8 mg/g) and the highest total flavonoid content (27.07 mg/g) were estimated for treatment No. 16, which consisted of 200 mg/L polyethylene glycol (4000) and exposure to 30 W UV for 60 min (PEG4). The fully grown callus produced by treatment No. 16 is shown in Figure 2. PEG4 increased the production level of the phenolic and flavonoid contents due to the elicitation effect caused by the high content of polyethylene glycol (4000), which is well known for its strong elicitation effect in the in vitro production of phenolics and flavonoids [37,38,39]. The exposure of the callus to ultraviolet radiation causes an elevation of the phenolic and flavonoid contents in many plants, such as *Lepidium sativum* L [40] and *Lactuca undulata* [41]. Coupling the physical and chemical elicitors enhances the chemical profile of the plant, as the physical elicitation can enhance the chemical profile of the callus as well as increase its biological activity [42]. In this study, the coupling of chemical and physical elicitors in PEG 4 caused an increase in the total phenolics and total flavonoids, which were 4.1 and 4.9 times those of the ME and 4.9 and 4.8 times those of the control sample, respectively.

#### 2.1.3. LC-ESI-MS/MS Profiling of Polyphenols in MRM Mode

The phenolic compounds were qualitatively and quantitatively identified in each treatment as well as in the control sample using liquid chromatography combined with electrospray ionization triple quadrupole mass spectrometry in MRM mode. The analysis parameters and conditions are described in the Materials and Methods section. The LC-ESI-MS/MS chromatogram of 21 phenolic and flavonoid standard compounds is represented in Figure S1. The LC-ESI-MS/MS chromatograms in MRM mode of phenolic and flavonoids of the different treatments are depicted in Figures S2–S17. The results of the analyses of the treatments differed in terms of the number of identified compounds and their concentration. The identified compounds and their concentration in each treatment are illustrated in Table 3.

Sixteen compounds were identified in PEG4, the most powerful treatment in terms of phenolic compound production: gallic acid, 3.4-dihydroxybenzoic acid, chlorogenic acid, caffeic acid, syringic acid, coumaric acid, vanillin, rutin, ellagic acid, ferulic acid, luteolin, quercetin, naringenin, kaempferol, apigenin, and hesperetin. The major compound was naringenin, with a concentration of 4922.71 µg/g dry weight, which represents 224.7 times the amount of naringenin in the control sample (21.99 µg/g dry weight). The concentration of the other identified compounds was also elevated in comparison to the control sample.

Generally, all the compound concentrations were increased in all treatments in comparison to the control. Furthermore, for each compound, there was a certain treatment that was the most productive. For the large-scale production of these identified compounds, we recommend choosing the most productive treatment for the targeted compound. The most productive treatment for each compound is illustrated in Table 4.

### 2.2. Molecular Docking Studies

**Figure 3:**
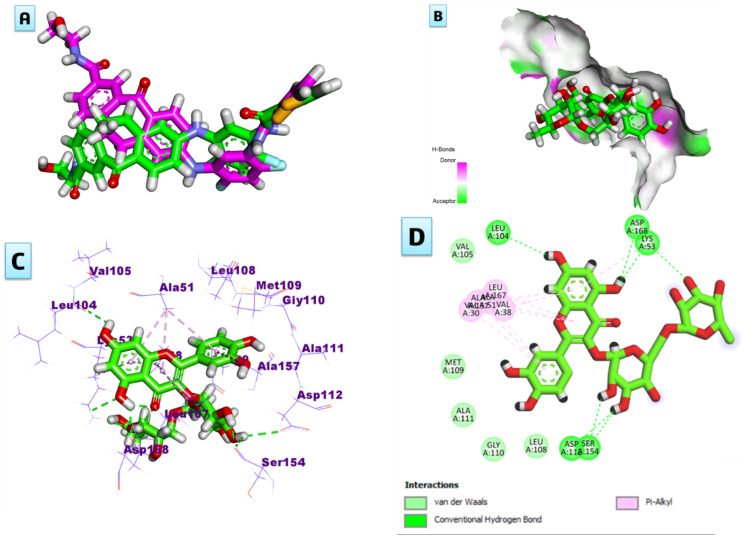
(**A**) Validation of the docking protocol, (**B**) surface map, (**C**) 3D binding mode, and (**D**) 2D binding mode of the rutin in the active site of the human p38*α* MAP kinase (PDB ID: 5MTY).

Docking studies of the identified compounds in the bioactive extract were performed as a theoretical attempt to find the enzymatic system that could be responsible for this activity. The results showed good binding affinities of the tested compounds to the catalytic domains of the human p38*α* MAP kinase with ΔG ^a^ ≥ 4.73 kcal/mol. The binding affinity of each compound is illustrated in Table 5, and rutin showed the highest binding affinity with ΔG ^a^ = 8.30 kcal/mol compared to the reference drug, doxorubicin (ΔG ^a^ = 7.43 kcal/mol). A careful inspection of the binding between the rutin and the active pocket of the protein revealed the presence of seven conventional hydrogen bonds, as shown in Figure 3. Among these hydrogen bonds, three were formed between the phenolic hydroxyls of the A-ring of the flavonol nucleus with the following crucial amino acids: Leu 104, Asp 168, and Lys 53. The other four bonds were formed between the alcoholic hydroxyls of the sugar moieties with Lys 53, Asp 112, and Ser 154. Additionally, the three aromatic rings of the flavonol exhibited π-alkyl interactions with the different amino acid residues of the active site, as shown in Figure 3.

### 2.3. The Anticancer Activity Assay Using MTT

**Figure 4:**
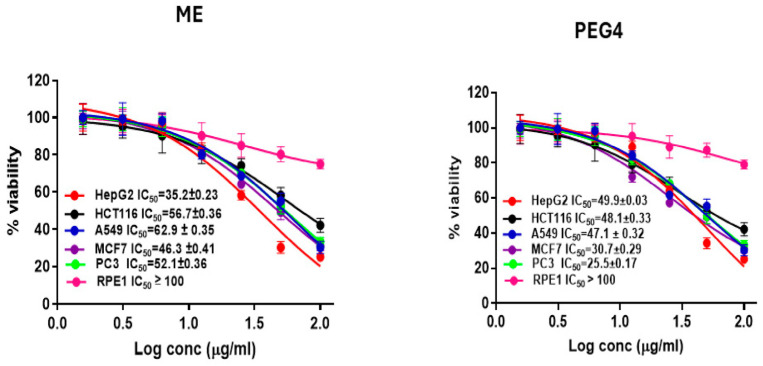
The cytotoxic effect of the 70% ethanolic extract of *Maesa indica* and the callus induced by it on PEG4 treatments.

The docking assay result encouraged us to establish an in vitro cytotoxic study of the ME and compare it with the cytotoxic effect of the tissue culture sample (PEG4). The cytotoxic effect of the plant cultivated in Egypt was investigated for the first time; furthermore, the cytotoxic effect of the callus produced by *Maesa indica* was investigated for the first time. The anticancer activity of the ME and PEG4 was tested by an MTT assay on five human cancer cell lines: hepatic (HepG2), prostate (PC3), lung (A549), breast (MCF7), and colon (HCT116), in addition to the normal human cell line, which was retinal pigment epithelial cells (RPE1). To evaluate the cytotoxic effect of the two samples, the concentration of the sample that caused 50% death in the cells after 48 h of treatment was calculated for each cell line using doxorubicin as a reference standard. The IC_50_ values are presented in Table 6 and Figure 4. The ME and PEG4 revealed cytotoxic effects on all five of the cell lines. PEG4 showed its highest cytotoxic effect againstPC3 with an IC_50_ of 25.5 µg/mL, followed by its effect against MCF7, with an IC_50_ of 30.7 µg/mL. PEG4’s lowest cytotoxic effect was against HepG2 with an IC_50_ of 49.9 µg/mL. On the other hand, the effect of the plant extract was lower than that of PEG4 on four cell lines (HCT116, MCF7, PC3, and A549). ME highest cytotoxic effect was on HePG2 with an IC_50_ of 35.2 µg/mL, and its lowest effect was on A549 with an IC_50_ of 62.9 µg/mL. The cytotoxic effect of PEG4 is significantly different from the effect of the plant extract on the five cell lines. PEG4’s cytotoxic effect on PC3 is about 2.1 times the cytotoxic effect of the ME, and its effect on MCF7 is about 1.5 times the effect of the ME. Its cytotoxic effect on A549 is about 1.3 times higher than the cytotoxic effect of the ME, while its effect on HePG2 is about 0.7 times the effect of the ME. The IC_50_ values of the ME and PEG4 on RPE1 were more than 100 µg/mL, which indicates that the two samples have a limited cytotoxic effect on normal cells. The cytotoxic effect is suggested to be due to the presence of phenolic compounds, which are well known for their cytotoxic effect on different cancer cell lines [43,44]. The higher polyphenolic content of PEG4 than that of the ME explains its higher cytotoxic effect. Furthermore, the identified compounds in the LC-ESI-MS/MS profiling of the polyphenols in MRM mode have been reported to have a cytotoxic effect. Gallic acid was revealed to have cytotoxic effects against hepatoma, promyelocytic leukemia, epidermoid carcinoma, and epithelial carcinoma [45], while another study reports its anticancer effect on MCF7 [46]. Naringenin was found to have an anticancer effect on hydrogen-peroxide-induced cytotoxicity and apoptosis in mouse leukemia P388 cells and has antioxidant and anti-apoptotic properties [47]. Naringenin possesses a strong cytotoxic effect against many different types of cancer, such as breast, liver, lung, prostate, pancreatic, uterine, brain, oral, neck, head, throat, skin, colon, bladder, bone, and carcinosarcoma cancers [48]. Chlorogenic acid showed cytotoxicity against oral tumors [49], human leukemia [50], osteosarcoma [51], breast cancer [52], lung cancer [53], colon cancer [54], and prostate cancer [55]. Vanillin can suppress breast [56], lung [57], and colorectal [58] cancers. Ferulic acid was proven to have anti-breast cancer [59], anti-lung cancer [60], anti-liver cancer [61], anti-colon cancer [62], and anti-prostate cancer [63] effects. The suggested cytotoxic activity of the samples could be due to the high content of the identified phenolic compounds, which were previously proven to have cytotoxic activity. The result of the current study is an encouraging step to start further in vivo and clinical trials of PEG4 to investigate its cytotoxic activity in humans.

## 3. Materials and Methods

### 3.1. Plant Material

Before blooming, *Maesa indica* Roxb. Sweet aerial parts were collected from El-Mazhar botanical garden in El-Barageel, Giza, Egypt. The plant was authenticated by Therese Labib, a senior botanist at the El-Orman Botanical Garden and a taxonomy consultant for the Egyptian Ministry of Agriculture. The specimen voucher had the serial number “19.06.2022” and was held in the herbarium of Cairo University’s Faculty of Pharmacy’s Pharmacognosy Department. The plant material was air-dried in the shade, pounded into a coarse powder, and kept in an appropriate airtight dark glass at 25 °C. Five hundred grams of the dried powdered *M. indica* aerial parts were extracted using three liters of 70% ethanol (three times until complete extraction) at room temperature. The ethanol was evaporated using a rotary evaporator apparatus (Buchi Rotavapor^®^ R-300) adjusted to 40 °C. The extracts were concentrated until complete dryness, yielding 5.8 gm of thick extract (ME).

### 3.2. Tissue Culture Study

#### 3.2.1. Explant Surface Sterilization

Leaves of *M. indica* were sterilized by dipping them in soapy water containing scepter soap, shaking them for 20 min, and then washing them in running tap water for 1 h. They were then immersed in 70% ethyl alcohol for 2 min, sterilized in 10% Clorox (NaOCl 5.25%) for 5 min, and then dipped in 0.1% (*w*/*v*) mercuric chloride with a few drops of Tween-20 for 15 min. Finally, the explants were rinsed at least three times with sterilized distilled water.

#### 3.2.2. Culture Initiation and Incubation Conditions

Full-strength MS media [64] supplemented with 2.5% (*w*/*v*) sucrose, 0.7% agar, and 0.25 gm/L casein hydrolysate [65] were prepared. The prepared media were divided into 18 treatments, each of which was enriched with different plant growth regulators (auxins and cytokines), then kept in Pyrex jars with 25 mL of media in each jar. Each treatment consisted of 8 jars (n = 8). The different treatments are illustrated in Table S2. Four sterilized explants (about 0.5–1 cm leaf fragments) were carried out from the mother plant and incubated on the different enhanced MS media using sterile scalpels and forceps under aseptic conditions. The photon-rich area of the incubation chamber was used for plant culture incubation at 25 ± 2 °C and under 40 μmol m^−2^s^−1^ photosynthetic photon flux density (PPFD) for 8 h in the dark and 16 h in the light for 4–6 weeks. The type of light source was cool white fluorescent tubes [66]. After 6 weeks, the fresh callus weight was determined for each treatment. MS-free treatment medium (free of growth hormones) was used as a control sample.

#### 3.2.3. Secondary Metabolite Production Elicitation

Four consecutive subcultures were made to increase the callus mass production on the most powerful medium in terms of callus induction, No. 16, before starting the secondary metabolite production step. In vitro secondary metabolite production was enhanced using chemical elicitation, physical elicitation, and coupling of chemical and physical elicitations (including UV-C light, sucrose, sorbitol, H_2_O_2_, and polyethylene glycol (4000)) with different concentrations and combinations [67]. Full-strength MS media supplemented with 2.5% (*w*/*v*) sucrose and 0.7% agar were prepared as basal media which were enriched with the different chemical elicitors before the pH was adjusted to 5.5–5.8 and all jars were sterilized using the autoclave. The calli were cultured as one gram on 25 mL of the medium in Pyrex glass jars, and each treatment consisted of 8 jars (n = 8). The physical elicitation was performed using two UV-C lamps (Philips TUV-15 W, 254 nm wavelength), and jars were exposed to the 30 W UV-C light from distances of 10 cm for 60 min. The control treatment, to which no elicitors were added, was used as a reference. The used elicitors in the different treatments are presented in Table 2. After an incubation period of 6 weeks, calli were spread on filter paper to remove excess water and dried in an oven for 2 h at 30 °C, powdered in a mortar, and then extracted using 70% ethanol. The dry callus extract was obtained by evaporating the solvent using a rotary evaporator apparatus (Buchi Rotavapor^®^ R-300) at 40 °C. To assess the secondary metabolite production capacity, total phenolics and flavonoids in each treatment were spectrophotometrically determined in one gram of the dry extract of callus. The dried 70% ethanolic extract of the parent plant aerial parts, ME, was also investigated for its total phenolics and flavonoids. Total flavonoids were expressed as mg catechin equivalents per gram of dry extract of the callus (mg CE/g), and total phenolics were expressed as mg gallic acid equivalents per gram of dry extract of the callus (mg GAE/g).

#### 3.2.4. Total Phenol and Total Flavonoid Content

The total phenolic content was measured according to the Folin–Ciocalteu method [68]. Briefly, the extract (100 µL) was transferred into a test tube, and the volume was adjusted to 3.5 mL with distilled water and oxidized with the addition of 250 µL of Folin–Ciocalteau reagent. After 5 min, the mixture was neutralized with 1.25 mL of 20% aqueous sodium carbonate (Na_2_CO_3_) solution. After 40 min, the absorbance was assessed at 725 nm against the blank. The total phenolic content was determined utilizing a calibration curve prepared with gallic acid and expressed as milligrams of gallic acid equivalent (mg GAE) per gram of the dry extract of the callus. Further dilution was performed in case the absorbance value measured was over the linear range of the standard curve.

The total flavonoid content was determined according to [68] using an aluminum chloride (AlCl_3_) colorimetric assay. Briefly, 300 µL of 5% sodium nitrite (NaNO_2_) was mixed with 100 µL of extract. After 6 min, 300 µL of a 10% AlCl_3_ solution was added, and the volume was adjusted to 2.5 mL using distilled water. After about 7 min, 1.5 mL of 1 M NaOH was gradually added, then the mixture was centrifuged for 10 min at 5000× *g*. The supernatant absorbance was assessed at 510 nm against the blank. The total flavonoid content was measured utilizing a calibration curve using catechin and expressed as milligrams of catechin equivalent (mg CE) per gram of the dry extract of the callus. An additional dilution was performed when the absorbance value exceeded the linear range of the standard curve.

#### 3.2.5. Statistical Analysis

All samples were determined in triplicate, and the results are expressed as mean ± S.D. The analysis variance (one-way ANOVA) was followed by Tukey’s multiple comparisons test to determine the significance of the difference between the control sample and the treatments. The statistical analysis was tested at *p* < 0.5 level of probability using GraphPad Prism version 6 (GraphPad Software Inc., San Diego, CA, USA).

### 3.3. LC-ESI-MS/MS-MRM Profiling of Polyphenols

#### 3.3.1. Chemicals and Instruments

The standard phenolic acids and flavonoids used in this study were purchased from Sigma. Formic acid (LC grade) was supplied from Fisher Chemical. Methanol and acetonitrile (LC grade) were purchased from Supelco. Milli-Q water was used. LC-MS/MS was performed using the ExionLC™ AC system coupled with AB Sciex Triple Quadruple 5500+ mass spectrometer. Electron spray ionization (ESI) was used, and the analysis was performed using the multiple reaction monitoring (MRM) mode. MRM transitions and the optimized mass spectrometer parameters of each compound are listed in Table S1.

#### 3.3.2. Chromatographic Conditions

The separation was performed using ZORBAX Eclipse Plus C18 Column (4.6 × 100 mm, 1.8 µm). The mobile phase consisted of two eluents: A: 0.1% formic acid in water; and B: acetonitrile (LC grade). The mobile-phase elution was programmed as follows: 2% B from 0–1 min, 2–60% B from 1–21 min, 60% B from 21–25 min, and 2% B from 25.01–28 min. The assay flow rate was adjusted at 0.8 mL/min while the injection volume was 3 µL. For the MRM analysis of the standard polyphenols, the positive and negative ionization modes were performed in the same run with the following parameters: curtain gas: 25 psi; ion spray voltage at 4500 and −4500 for positive and negative modes, respectively; source temperature: 400 °C; and collision energy spread: 10.

#### 3.3.3. Preparation of Standard Solutions

Stock standard solutions (1 mg/mL) of 21 standard phenolic compounds (gallic acid, caffeic acid, rutin, coumaric acid, vanillin, naringenin, quercetin, ellagic acid, 3.4-dihydroxybenzoic acid, hesperetin, myricetin, cinnamic acid, methyl gallate, kaempferol, ferulic acid, syringic, apigenin, catechin, luteolin, chlorogenic acid, and daidzein) were prepared in methanol. The stock solutions of each standard were further diluted to 10 μg/mL and kept at 4 °C. Working standard mixture solution (200 ppb) was prepared by mixing 20 μL of each stock solution and adding methanol until a volume of 1 mL was reached.

#### 3.3.4. Sample Preparation

A total of 3–5 mg of the dried extract of callus was dissolved in 1 mL 80% methanol, sonicated for 15 min, filtered through a 0.45 um syringe filter, and finally injected into LC-MS/MS. A blank (80% methanol) was injected before each sample.

#### 3.3.5. Peak Identification

The peak identification was performed according to the inserted parent ions (Q1), product ions (Q3), the retention time of the standard phenolic compounds that were previously injected, and the reported data in the literature.

#### 3.3.6. Quantitative Analysis

For quantitative analysis of polyphenolic compounds, the MS parameters were operated in MRM mode using precursor ions [M − H]^−^ and [M + H]^+^ and their product ions. The standards at various concentrations were used for the preparation of the calibration curves. The curves were prepared by linear regression based on peak area and the mass parameter.

### 3.4. Molecular Docking Studies

The molecular docking studies of the compounds of the extracts, identified using the LC-ESI-MS/MS-MRM technique, have been carried out via Molecular Operating Environment (MOE) software version 2015.10 [69] at the catalytic domains of the human p38α MAP kinase (PDB ID: 5MTY) [70]. From the Protein Data Bank (PDB), the suitable conformation of the human p38α MAPK was obtained. After removing the undesirable solvents and cofactors, the protein was prepared as described previously [71]. The appropriate binding pockets containing the crucial amino acid residues were identified using the “Site Finder” feature. The reference drug, doxorubicin, and the tested compounds underwent protonation and energy minimization. Re-docking the co-crystallized ligand in the active binding site was performed to validate the technique. Subsequently, the tested compounds and the reference database files (MDB) were docked into the protein’s binding site. The results of the process are shown as ΔG ^a^ (kcal/mol) values, which are the binding free energies that give an idea of how preferable a certain docking pose is. These scoring functions use different non-covalent types of force fields to predict protein–ligand interactions. The best poses of the compounds with the highest ΔG ^a^ and RMSD values ≤2 Å were obtained in 2D and 3D representations.

### 3.5. Cytotoxic Effect on Human Cell Lines

The MTT method was used to measure cellular metabolic activity to explore the cell viability, proliferation, and cytotoxicity. In this colorimetric measurement, the principle is the reduction of a yellow tetrazolium salt (3-(4,5-dimethylthiazol-2-yl)-2,5- diphenyltetrazolium bromide or MTT) to purple crystals of formazan by metabolically active cells [72]. Using a laminar flow cabinet of biosafety class II (Baker, SG403INT, Sanford, ME, USA), all of the following operations were carried out in a sterile environment. Cells were suspended in a 1% antibiotic-antimitotic mixture (10,000 U/mL potassium penicillin, 10,000 µg/mL streptomycin sulfate, and 20 µg/mL amphotericin B), 1% L-glutamine, and RPMI 1640 media for five human cancer cell lines: HepG2 (liver cancer cell line), PC3 (prostate cancer cell line), A549 (lung cancer cell line), MCF7 (breast cancer cell line), and HCT116 (colon cancer cell line), in addition to RPE (normal human retinal pigment epithelial cell line) at 37 °C under 5% CO_2_. Cells were batch-grown for ten days, after which they were seeded in 96-well microtiter plastic plates at a concentration of 10 × 10^3^ cells/well in new complete growth media at 37 °C for twenty-four hours under 5% CO_2_ using a water-jacketed carbon dioxide incubator (Sheldon, TC2323, Cornelius, OR, USA). Media were aspirated, fresh medium was added, and cells were cultured either alone (negative control) or with varied concentrations of the tested samples, ME and PEG4 (100, 50, 25, 12.5, 6.25, 3.125, 0.78 and 1.56 μg/mL). The medium was aspirated after 48 h of incubation, 40 µL of MTT salt (2.5 μg/mL) was added to each well, and the incubation process was continued for a further four hours at 37 °C. A volume of 200 µL of 10% sodium dodecyl sulfate (SDS) in deionized water was added to each well and incubated overnight at 37 °C to terminate the reaction and dissolve the crystals that had formed. Different concentrations of doxorubicin (0.01–100 µg/mL) were used as a positive control. According to studies by [73,74], doxorubicin is utilized as a recognized cytotoxic natural substance that produces 100% lethality under identical conditions at a concentration of 100 µg/mL. Next, the absorbance was determined at 595 nm using a reference wavelength of 620 nm and a microplate multi-well reader (Bio-Rad Laboratories Inc., model 3350, Hercules, CA, USA). The samples were determined in triplicate, and the results are expressed as mean ± S.D. Using one-way ANOVA followed by Tukey’s multiple comparisons test, a statistical significance was examined between the samples and the negative control at *p* < 0.5 level of probability. Plant extracts are dissolved in DMSO, which has a final concentration of less than 0.2% in the cells. The following method was used to determine the percentage change in viability: ((extract reading/negative control reading) − 1) × 100. Measurements of the cytotoxic effect of the two samples depended on the calculation of the concentration that caused 50% death in the cells after 48 h of treatment, IC_50_, for each cell line, and doxorubicin was used as the reference standard.

## 4. Conclusions

In this study, a protocol for callus induction from *Maesa indica* was established for the first time. The MS medium supplemented with 0.25 gm/L casein hydrolysate and 4 mg/L picloram exhibited the highest callus production capacity. In addition, 16 different chemical and physical elicitation treatments were used to enhance the phytochemical profile of the calli. The total phenolics and total flavonoids of the different treatments were measured, and the phenolic and flavonoid compounds were qualitatively and quantitatively determined using LC-ESI-MS/MS-MRM analyses. Docking studies were performed for the identified compounds, and a cytotoxic study against five human cancer cell lines was performed. The elicitation protocol caused an elevation in the total phenolics and total flavonoids in all treatments, and PEG4 had the highest contents of these. The elicitation in PEG4 caused an elevation of the total phenolics and total flavonoids, which were 4.1 and 4.9 times higher in comparison with those of the ME and 4.9 and 4.8 times higher in comparison with those of the control sample, respectively. LC-ESI-MS/MS-MRM was used for the qualitative and quantitative determination of phenolic compounds in the 16 treatments. The identified compounds’ concentrations were elevated after the elicitation, especially those in PEG4. The docking of the tested compounds showed good binding affinities to the catalytic domains of the human p38*α* MAP kinase, and rutin showed the highest binding affinity. Both ME and PEG4 are suggested to have cytotoxic effects on five cell lines. PEG4 has its highest cytotoxic effect against PC3 followed by MCF7. PEG4’s cytotoxic effect on PC3 is about 2.1 times the cytotoxic effect of the ME, and its effect on MCF7 is about 1.5 times the effect of theME. Further in vivo and clinical studies are required to explore the cytotoxic activity of the plant and the induced callus.

## Figures and Tables

**Table 1:** The average weights of calli induced by different treatments.

Treatment No.	Media Composition	The Average Weight in gm ± SD.
1	Control (MS-free treatment)	1.06 ± 0.095
2	MS + 0.25 gm/L CH + 1 mg/L 2,4-D + 0.33 /L 2-iP	2.6 ± 0.201 *
3	MS + 0.25 gm/L CH + 1 mg/L 2,4-D + 0.33 mg/L BAP	2.85 ± 0.101 *
4	MS + 0.25 gm/L CH + 2 mg/L 2,4-D	2.3 ± 0.0867 *
5	MS + 0.25 gm/L CH + 2 mg/L 2,4-D + 0.33 mg/L 2-iP	2.7 ± 0.012 *
6	MS + 0.25 gm/L CH + 2 mg/L 2,4-D + 0.33 mg/L BAP	2.75 ± 0.132 *
7	MS + 0.25 gm/L CH + 4 mg/L 2,4-D	2.5 ± 0.113 *
8	MS + 0.25 gm/L CH + 4 mg/L 2,4-D + 0.33 mg/L 2-iP	1.96 ± 0.198 *
9	MS + 0.25 gm/L CH + 4 mg/L 2,4-D + 0.33 mg/L BAP	2.1 ± 0.097 *
10	MS + 0.25 gm/L CH + 1 mg/L Picloram	2.94 ± 0.158 *
11	MS + 0.25 gm/L CH + 1 mg/L Picloram + 0.33 mg/L 2-Ip	3.2 ± 0.145 *
12	MS + 0.25 gm/L CH + 1 mg/L Picloram + 0.33 mg/L BAP	2.4 ± 0.22 *
13	MS + 0.25 gm/L CH + + 2 mg/L Picloram	3.8 ± 0.172 *
14	MS + 0.25 gm/L CH + 2 mg/L Picloram + 0.33 mg/ L 2-iP	2.1 ± 0.741
15	MS + 0.25 gm/L CH + 2 mg/L Picloram + 0.33 mg/ L BAP	3.2 ± 0.0874 *
16	MS + 0.25 gm/L CH + 4 mg/L Picloram	7.5 ± 0.854 *
17	MS + 0.25 gm/L CH +4 mg/L Picloram + 0.33 mg/L 2-iP	4.1 ± 0.147 *
18	MS + 0.25 gm/L CH + 4 mg/L Picloram + 0.33 mg/L BAP	3.8 ± 0.658 *

MS = Murashige and Skoog media. 2,4-D = 2,4 Dichlorophenoxy acetic acid. BAP = Benzyl amino purine. 2IP = 2-iso-pentenyl adenine. CH = Casein hydrolysate. Data are expressed as mean ± SD (n = 3). Statistical analysis was performed using one-way ANOVA followed by Tukey’s multiple comparisons test. * Significant difference from the control sample at *p* ˂ 0.01.

**Table 2:** Effect of different elicitors on the total phenolic and flavonoid content of *Maesa indica* calli.

No.	Treatment	Total Phenolics (mg GAE/g Dry Extract) Means ± SD	Total Flavonoids (mg CE/gm Dry Extract) Means ± SD
1	Control	9.88 ± 0.81	5.59 ± 0.36
2	30 W UV for 60 min.	40.14 ± 1.14 *	20.12 ± 0.21 *
3	10 mM H_2_O_2_	45.52 ± 0.72 *	25.14 ± 0.26 *
4	10 mM H_2_O_2_ + 30 W UV for 60 min.	26.15 ± 0.48 *	10.46 ± 0.19 *
5	Sucrose 25 gm /L	15.49 ± 0.21 *	6.51 ±0.19 *
6	Sucrose 25 gm/L+ 30 W UV for 60 min.	19.89 ± 0.30 *	10.34 ± 0.16 *
7	Sucrose 50 gm/L	11.67 ± 0.25 *	5.02 ± 0.11 *
8	Sucrose 50 gm/L + 30 W UV for 60 min.	8.62 ± 0.20 *	3.62 ± 0.08 *
9	Sorbitol 25 gm/L	29.38 ± 0.34 *	13.52 ± 0.15 *
10	Sorbitol 25 gm/L + 30 W UV for 60 min.	11.99 ± 0.11 *	5.76 ± 0.05 *
11	Sorbitol 50 gm/L	16.52 ± 0.27 *	8.09 ± 0.13 *
12	Sorbitol 50 gm/L + 30 W UV for 60 min.	16.54 ± 0.28 *	8.81 ±0.08 *
13	Polyethylene glycol (4000) 100 mg/L	6.30 ± 0.39 *	3.31 ± 0.06 *
14	Polyethylene glycol (4000) 100 mg/L + 30 W UV for 60 min.	28.60 ± 0.22 *	16.02 ± 0.12 *
15	Polyethylene glycol (4000) 200 mg/L	27.47 ± 0.53 *	14.44 ± 0.06 *
16	Polyethylene glycol (4000) 200 mg/L + 30 W UV for 60 min. (PEG4)	48.82 ± 0.44 *	27.07 ± 0.21 *
17	Mother plant extract	11.83 ± 0.22 *	5.44 ± 0.10 *

Data are expressed as mean ± SD (n = 3). Statistical analysis was performed using one-way ANOVA followed by Tukey’s multiple comparisons test. * Significant difference from the control sample at *p* < 0.01.

**Table 3:** Concentrations of identified flavonoids and phenolics expressed as µg/g dry powder of different calli.

No.	Compound	RT (min)	RRT	Conc. (µg/g)								
				#1	#2	#3	#4	#5	#6	#7	#8	#9
1	Gallic acid	3.81	0.39	10.81	83.91	156.64	44.07	103.74	43.34	42.95	36.05	11.64
2	3.4-Dihydroxybenzoic acid	5.72	0.58	41.79	801.55	1217.96	923.78	1108.87	336.31	426.20	274.81	350.75
3	Chlorogenic acid	7.34	0.74	10.03	174.25	262.75	25.20	173.71	315.92	99.36	57.03	153.00
4	Catechin	7.34	0.74	ND	19.29	38.34	ND	ND	ND	ND	ND	38.76
5	Methyl gallate	7.45	0.75	0.20	ND	ND	ND	ND	ND	ND	ND	ND
6	Caffeic acid	8.04	0.81	23.66	46.82	63.71	ND	47.53	21.81	16.43	16.74	7.95
7	Syringic acid	8.4	0.85	10.77	197.51	190.16	129.56	224.24	113.24	78.67	83.18	54.69
8	Coumaric acid	9.52	0.96	8.39	54.86	94.15	47.40	51.85	20.40	15.32	28.19	11.97
9	Vanillin	9.56	0.97	14.50	738.20	822.08	1029.93	794.45	410.36	324.71	290.04	116.29
10	Rutin	9.7	0.98	2.85	6.33	7.80	3.01	4.73	3.74	3.89	1.01	1.10
11	Ellagic acid	9.9	1	2.67	40.19	43.43	23.27	71.90	11.59	11.89	10.73	7.16
12	Ferulic acid	10.23	1.03	13.59	182.43	275.22	77.08	205.36	97.17	52.85	91.58	39.71
13	Myricetin	11.7	1.18	ND	77.96	ND	ND	ND	ND	ND	ND	ND
14	Daidzein	12.91	1.3	ND	ND	ND	ND	ND	ND	11.94	9.77	6.15
15	Luteolin	13.5	1.36	6.39	21.07	19.78	21.46	13.83	9.35	7.51	11.13	5.37
16	Quercetin	13.57	1.37	1.54	32.88	34.86	15.23	9.42	12.87	8.12	4.93	1.76
17	Cinnamic acid	14.18	1.43	ND	ND	ND	ND	ND	ND	ND	ND	ND
18	Naringenin	15.03	1.518	21.99	4486.30	3485.29	2378.72	465.57	3493.56	1585.68	1133.30	1407.17
19	Apigenin	15.04	1.519	ND	ND	ND	170.07	ND	57.19	86.96	65.02	ND
20	Kaempferol	15.34	1.55	4.23	8.99	9.97	5.95	6.40	6.16	1.51	2.02	1.25
21	Hesperetin	15.62	1.58	ND	2.66	3.42	16.91	5.39	3.44	1.22	2.02	1.33
**No.**	**Compound**	**RRT**	**RT (min)**	**Conc. (µg/g)**								
				**#10**		**#11**	**#12**	**#13**	**#14**		**#15**	**#16**
1	Gallic acid	0.39	3.81	20.86		8.34	5.24	299.49	233.16		53.66	103.32
2	3.4-Dihydroxybenzoic acid	0.58	5.72	281.39		120.52	102.04	3564.93	1749.80		2037.25	1621.39
3	Chlorogenic acid	0.74	7.34	248.12		40.00	73.05	46.88	104.82		264.53	1906.76
4	Catechin	0.74	7.34	24.66		36.24	42.14	47.48	24.62		68.28	ND
5	Methyl gallate	0.75	7.45	ND		ND	ND	ND	ND		ND	ND
6	Caffeic acid	0.81	8.04	24.23		11.78	8.30	18.85	51.50		66.00	56.60
7	Syringic acid	0.85	8.4	84.26		38.85	31.00	122.41	203.33		366.19	205.64
8	Coumaric acid	0.96	9.52	31.71		10.27	8.39	43.90	54.17		79.25	50.04
9	Vanillin	0.97	9.56	216.36		72.21	63.40	628.26	1049.94		943.53	960.10
10	Rutin	0.98	9.7	4.67		0.62	0.53	2.11	3.23		4.63	6.45
11	Ellagic acid	1	9.9	12.77		4.99	4.63	44.94	36.42		25.95	47.12
12	Ferulic acid	1.03	10.23	69.32		46.19	30.21	71.59	226.31		197.13	213.60
13	Myricetin	1.18	11.7	ND		ND	ND	ND	ND		ND	ND
14	Daidzein	1.3	12.91	15.04		3.39	2.96	3.76	18.76		ND	ND
15	Luteolin	1.36	13.5	13.21		5.03	3.24	2.17	12.37		15.18	36.82
16	Quercetin	1.37	13.57	8.05		5.49	3.45	2.21	13.82		16.38	38.14
17	Cinnamic acid	1.43	14.18	ND		ND	ND	ND	ND		ND	ND
18	Naringenin	1.518	15.03	1554.09		989.68	602.51	149.24	3906.75		4132.68	4922.71
19	Apigenin	1.519	15.04	79.36		29.64	29.92	33.13	117.60		96.51	127.92
20	Kaempferol	1.55	15.34	11.64		0.50	0.53	12.00	2.60		4.47	2.59
21	Hesperetin	1.58	15.62	3.06		0.95	1.09	1.11	0.32		24.01	3.91

# Treatment number. RT = Retention time. RRT = Relative retention time to ellagic acid. ND = Not determined.

**Table 4:** The most productive treatment for each identified compound.

Compound	The Most Productive Treatment	Compound	The Most Productive Treatment
Gallic acid	13	Ferulic acid	14
3.4-Dihydroxybenzoic acid	15	Myricetin	16
Chlorogenic acid	16	Ellagic acid	5
Catechin	15	Luteolin	16
Caffeic acid	15	Quercetin	16
Syringic acid	15	Naringenin	16
Coumaric acid	2	Apigenin	16
Vanillin	14	Kaempferol	13
Rutin	3	Hesperetin	14

**Table 5:** The binding affinities of the tested compounds to the catalytic domains of the human p38*α* MAP kinase with ΔG ^a^ ≥ 4.73 kcal/mol.

No.	Name	ΔG ^a^ (kcal/mol)
1	Apigenin	−5.90
2	Caffeic acid	−4.75
3	Catechin	−6.13
4	Chlorogenic acid	−6.92
5	Cinnamic acid	−4.73
6	(*E*)-*p*-coumaric acid	−4.95
7	Daidzein	−5.75
8	3,4-dihydroxybenzoic	−4.48
9	Ellagic acid	−4.95
10	Ferulic acid	−5.46
11	Gallic acid	−4.89
12	Hesperetin	−6.61
13	Kaempferol	−5.42
14	Luteolin	−6.24
15	Methyl gallate	−4.95
16	Myricetin	−6.04
17	Naringenin	−6.38
18	Quercetin	−5.59
19	Rutin	−8.30
20	Syringic acid	−5.09
21	Vanillin	−4.92
22	Co-crystallized ligand	−9.40
23	Doxorubicin	−7.43

**Table 6:** The cytotoxic effect of the ethanolic extract of *Maesa indica* and the callus induced by it on PEG4 treatments.

Cell Line	IC_50_ (µg/mL) Mean ± SD		
	ME	PEG4	Doxorubicin
HepG2	35.2 ± 0.23 *	49.9 ± 0.28 *^#^	5.8 ± 0.9
HCT116	56.7 ± 0.37 *	48.1 ± 0.31 *^#^	0.8 ± 0.02
A549	62.9 ± 0.58 *	47.1 ± 0.32 *^#^	4.3 ± 0.13
MCF7	46.3 ± 0.41 *	30.7 ± 0.29 *^#^	1.2 ± 0.14
PC3	52.1 ± 0.36 *	25.5 ± 0.17 *^#^	2.8 ± 0.14
RPE1	>100	>100	6.3 ± 0.58

Data areexpressed as mean ± S.E.M (n = 3). The significance of differences among the studied groups was determined using one-way ANOVA followed by Tukey’s multiple comparisons test. * Significant difference from the standard drug at *p* < 0.05. ^#^ Significant difference from ME at *p* < 0.05.

## Data Availability

The data presented in this study are available on request from the corresponding author.

## Supplementary Materials

The following supporting information can be downloaded at: https://www.mdpi.com/article/10.3390/plants13141979/s1, Figure S1. LC-MS/MS chromatogram obtained in MRM mode of a standard solution containing the analyzed polyphenols; Figure S2. LC-MS/MS chromatogram obtained in MRM mode of 70% ethanolic extract of treatment no. 1 callus; Figure S3. LC-MS/MS chromatogram obtained in MRM mode of 70% ethanolic extract of treatment no. 2 callus; Figure S4. LC-MS/MS chromatogram obtained in MRM mode of 70% ethanolic extract of treatment no. 3 callus; Figure S5. LC-MS/MS chromatogram obtained in MRM mode of 70% ethanolic extract of treatment no. 4 callus; Figure S6. LC-MS/MS chromatogram obtained in MRM mode of 70% ethanolic extract of treatment no. 5 callus; Figure S7. LC-MS/MS chromatogram obtained in MRM mode of 70% ethanolic extract of treatment no. 6 callus; Figure S8. LC-MS/MS chromatogram obtained in MRM mode of 70% ethanolic extract of treatment no. 7 callus; Figure S9. LC-MS/MS chromatogram obtained in MRM mode of 70% ethanolic extract of treatment no. 8 callus; Figure S10. LC-MS/MS chromatogram obtained in MRM mode of 70% ethanolic extract of treatment no. 9 callus; Figure S11. LC-MS/MS chromatogram obtained in MRM mode of 70% ethanolic extract of treatment no. 10 callus; Figure S12. LC-MS/MS chromatogram obtained in MRM mode of 70% ethanolic extract of treatment no. 11 callus; Figure S13. LC-MS/MS chromatogram obtained in MRM mode of 70% ethanolic extract of treatment no. 12 callus; Figure S14. LC-MS/MS chromatogram obtained in MRM mode of 70% ethanolic extract of treatment no. 13 callus; Figure S15. LC-MS/MS chromatogram obtained in MRM mode of 70% ethanolic extract of treatment no. 14 callus; Figure S16. LC-MS/MS chromatogram obtained in MRM mode of 70% ethanolic extract of treatment no. 15 callus; Figure S17. LC-MS/MS chromatogram obtained in MRM mode of 70% ethanolic extract of treatment no. 16 callus; Table S1. The multiple reaction monitoring transitions and the optimized mass spectrometer parameters; Table S2. Different plant growth regulators are used for *M. indica* callus induction.
