# Supplementary material for: Eliciting Callus Cultures for the Production of Cytotoxic Polyphenolics from Maesa indica Roxb. Sweet
Source: Plants (Basel). 2024 Jul 19;13(14):1979. doi: 10.3390/plants13141979 (PMC11280962; doi:10.3390/plants13141979)
Supplement: Supplementary file 1 [file plants-13-01979-s001.zip › plants-3076541-supplementary.pdf]

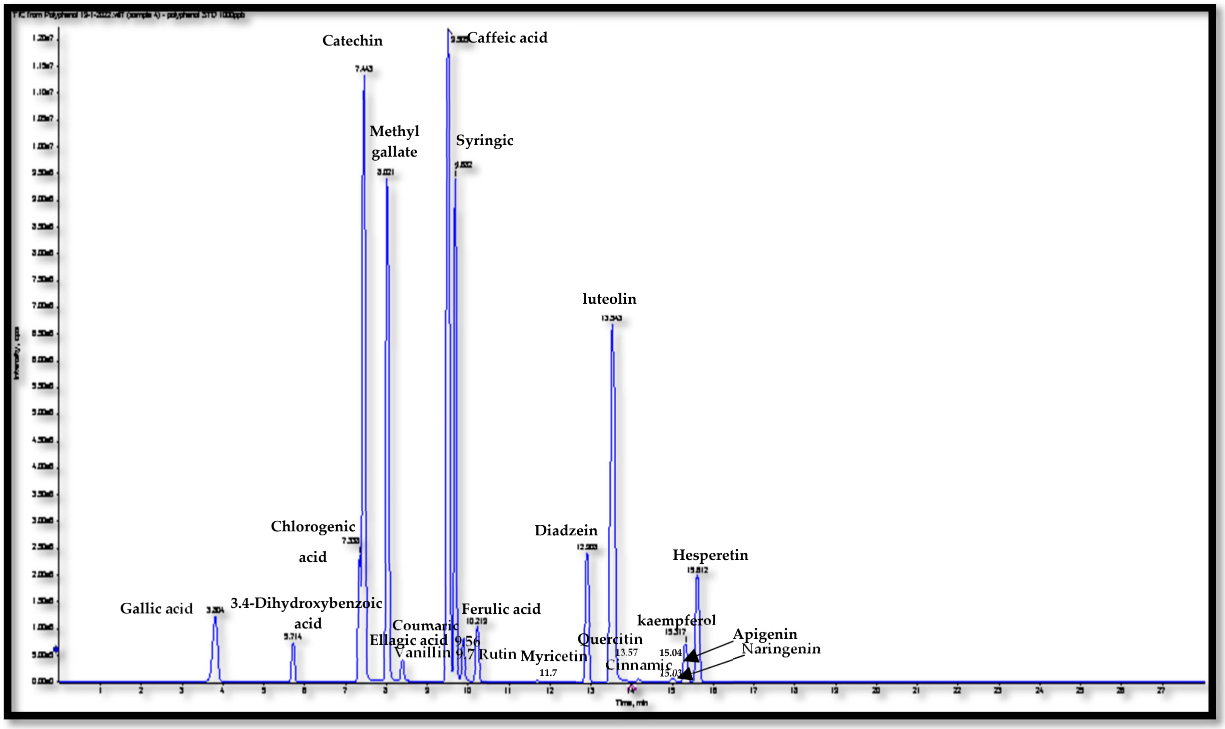

Figure S1. LC-MS/MS chromatogram obtained in MRM mode of a standard solution containing 21 polyphenolic compounds.

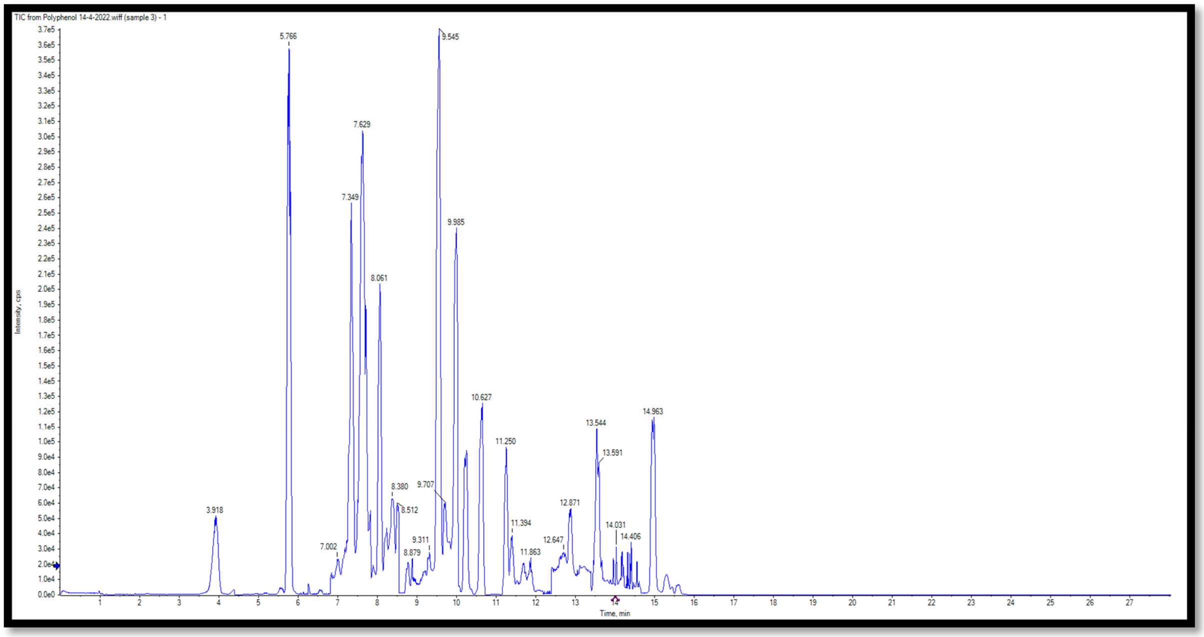

Figure S2. LC-MS/MS chromatogram obtained in MRM mode of 70% ethanolic extract of treatment no. 1 callus

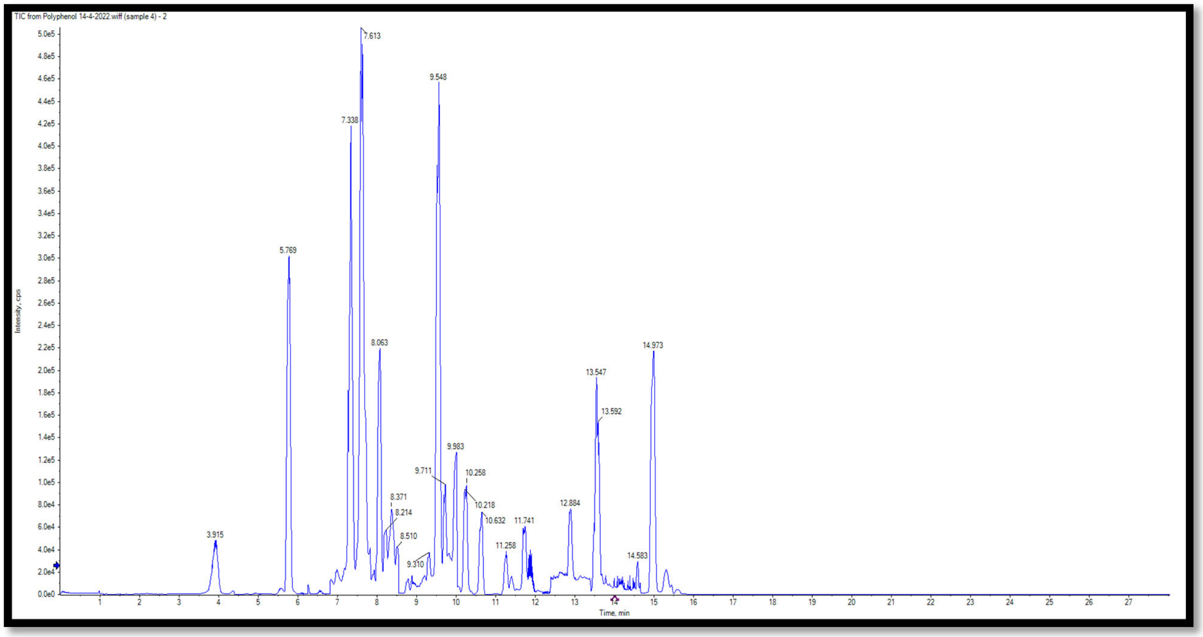

Figure S3. LC-MS/MS chromatogram obtained in MRM mode of 70% ethanolic extract of treatment no. 2 callus.

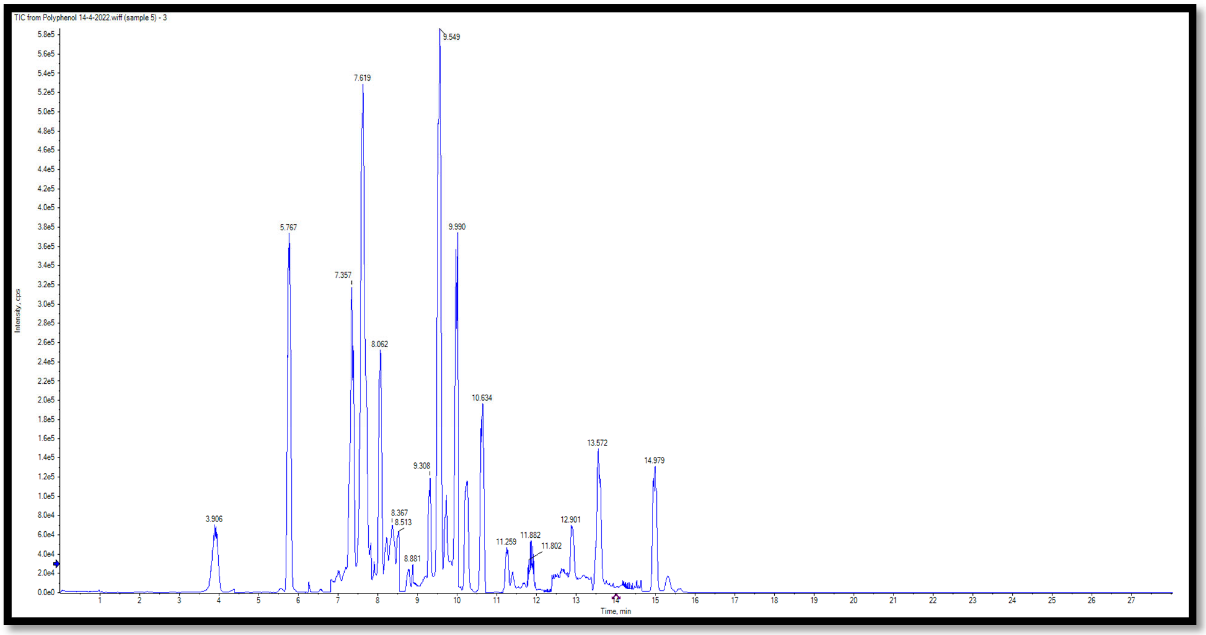

Figure S4. LC-MS/MS chromatogram obtained in MRM mode of 70% ethanolic extract of treatment no. 3 callus.

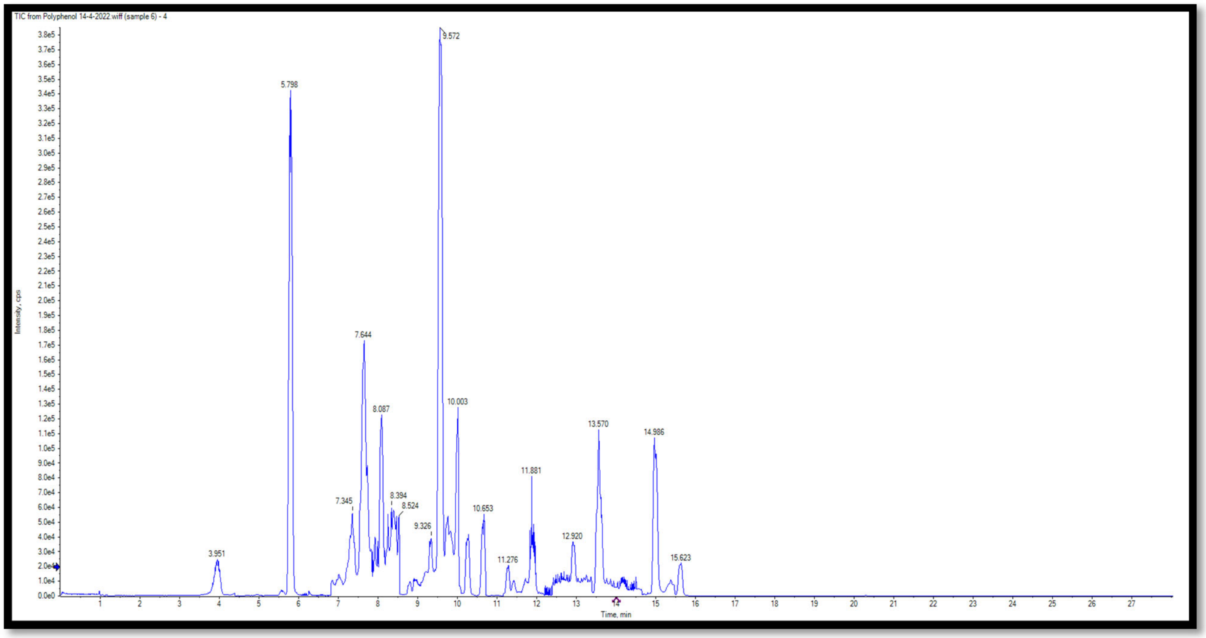

Figure S5. LC-MS/MS chromatogram obtained in MRM mode of 70% ethanolic extract of treatment no. 4 callus.

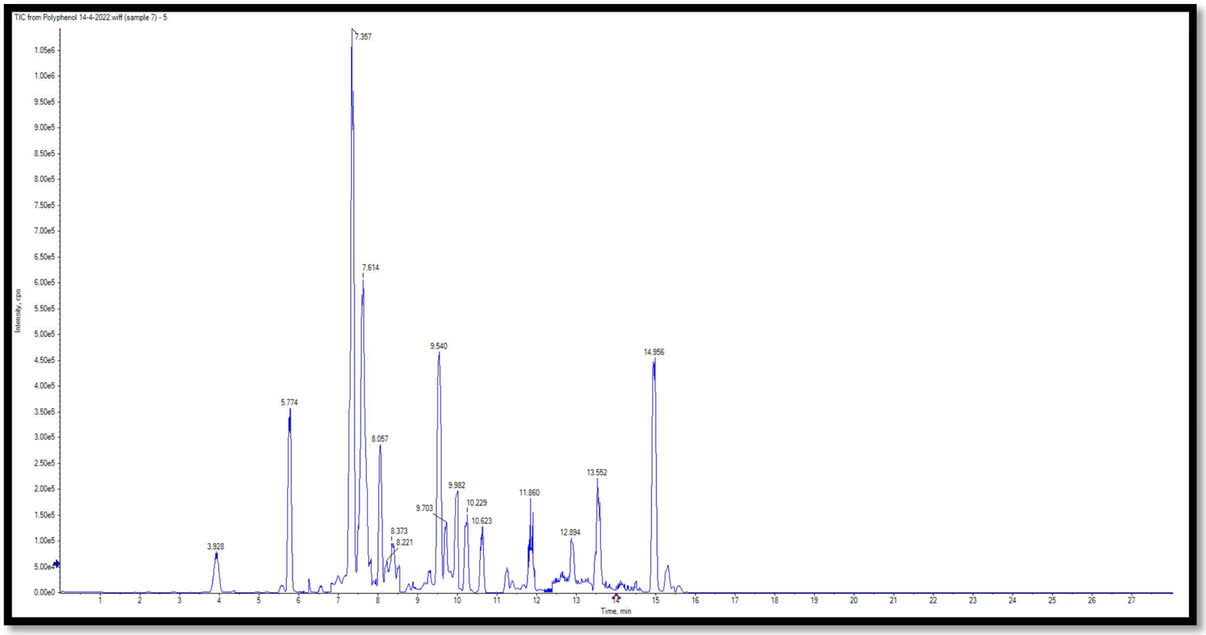

Figure S6. LC-MS/MS chromatogram obtained in MRM mode of 70% ethanolic extract of treatment no. 5 callus.

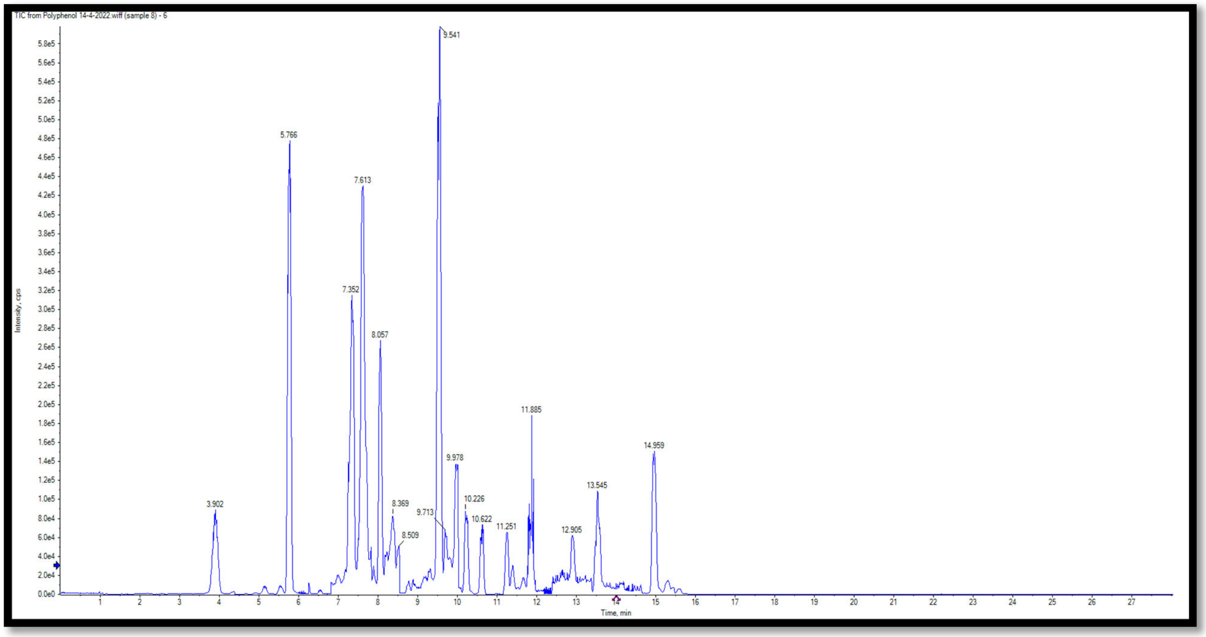

Figure S7. LC-MS/MS chromatogram obtained in MRM mode of 70% ethanolic extract of treatment no. 6 callus.

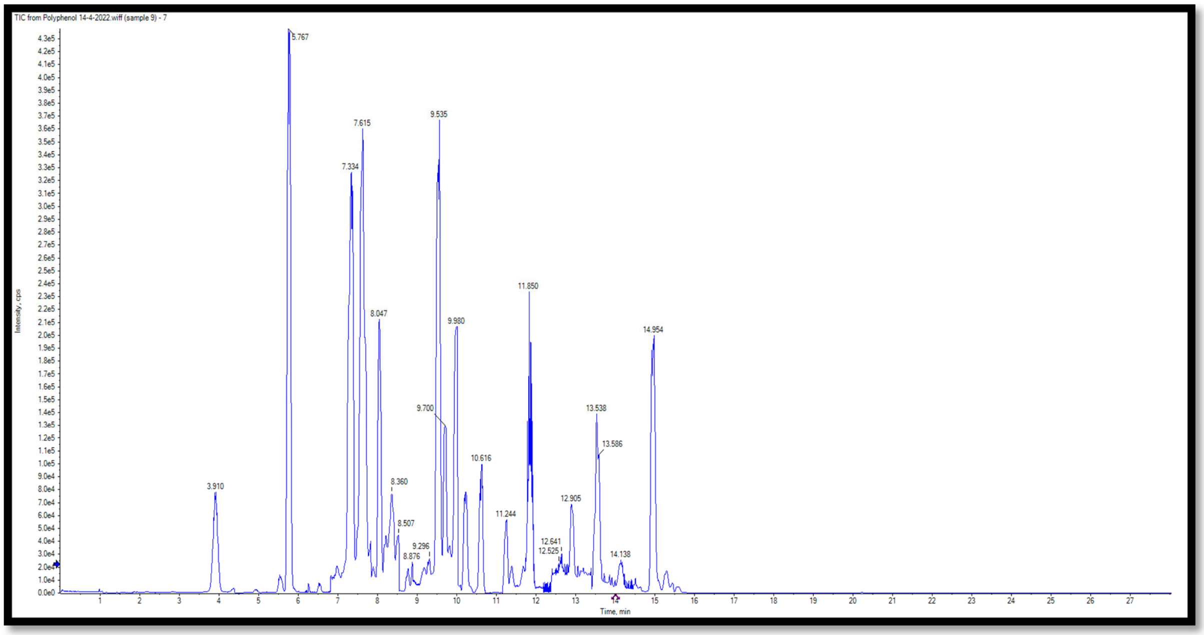

Figure S8. LC-MS/MS chromatogram obtained in MRM mode of 70% ethanolic extract of treatment no. 7 callus.

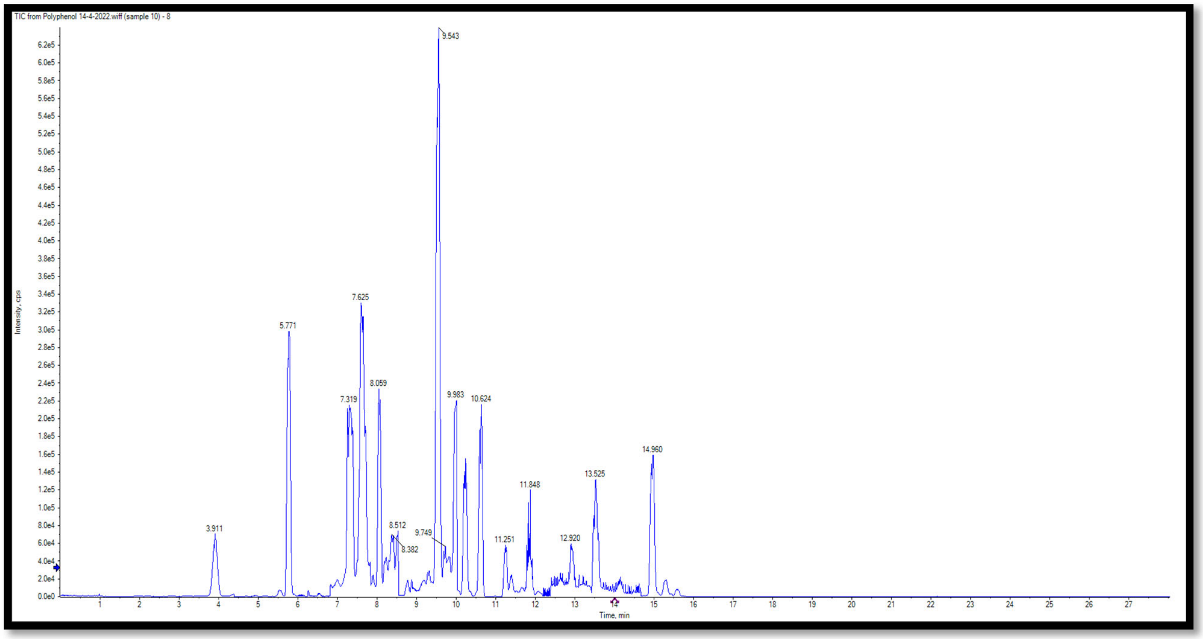

Figure S9. LC-MS/MS chromatogram obtained in MRM mode of 70% ethanolic extract of treatment no. 8 callus.

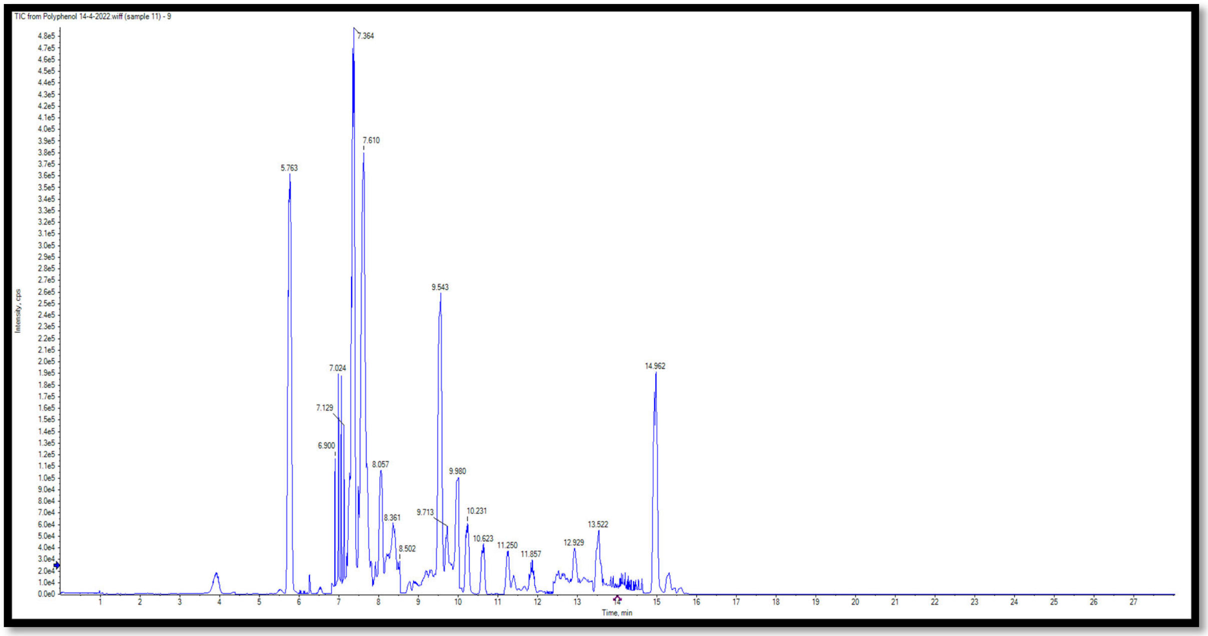

Figure S10. LC-MS/MS chromatogram obtained in MRM mode of 70% ethanolic extract of treatment no. 9 callus.

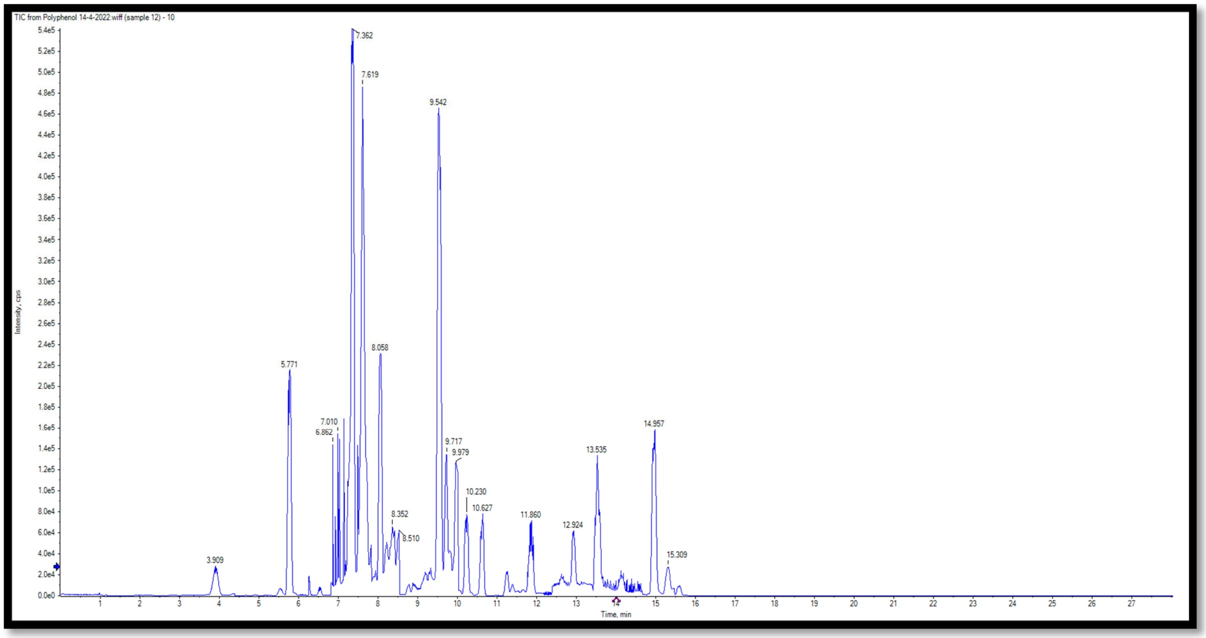

Figure S11. LC-MS/MS chromatogram obtained in MRM mode of 70% ethanolic extract of treatment no. 10 callus.

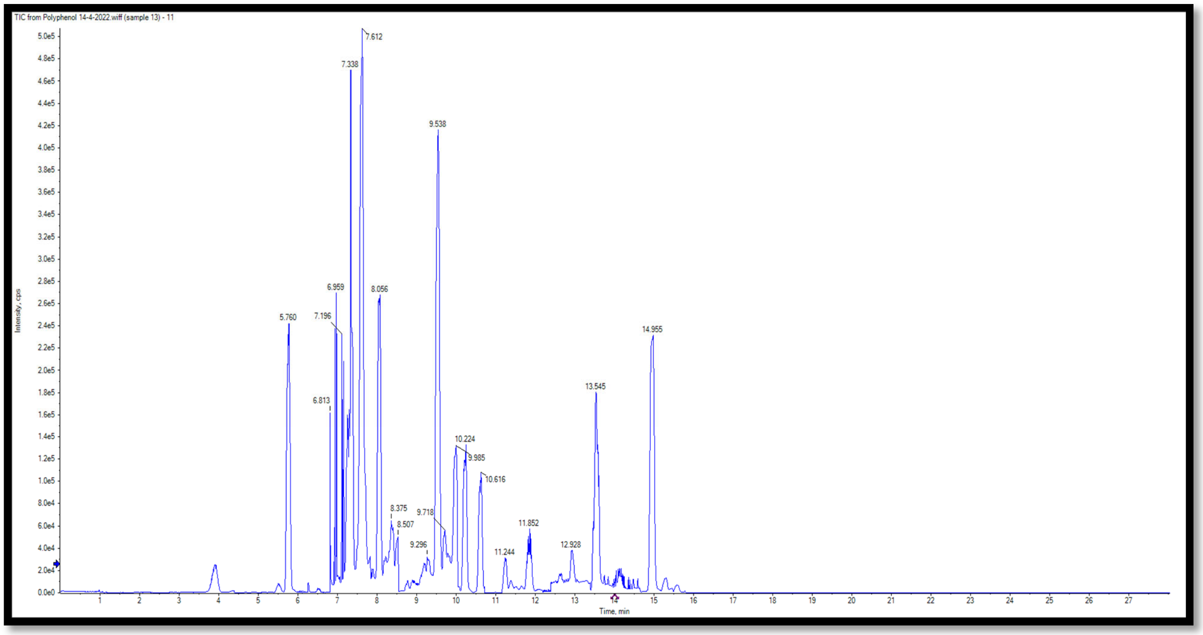

Figure S12. LC-MS/MS chromatogram obtained in MRM mode of 70% ethanolic extract of treatment no. 11 callus.

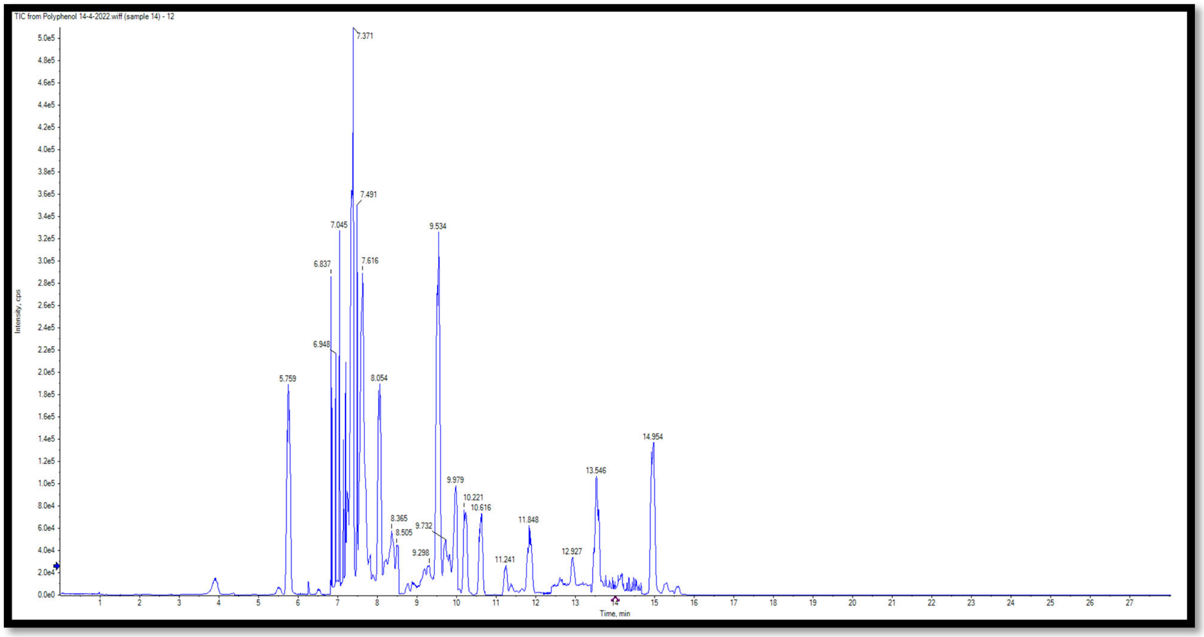

Figure S13. LC-MS/MS chromatogram obtained in MRM mode of 70% ethanolic extract of treatment no. 12 callus.

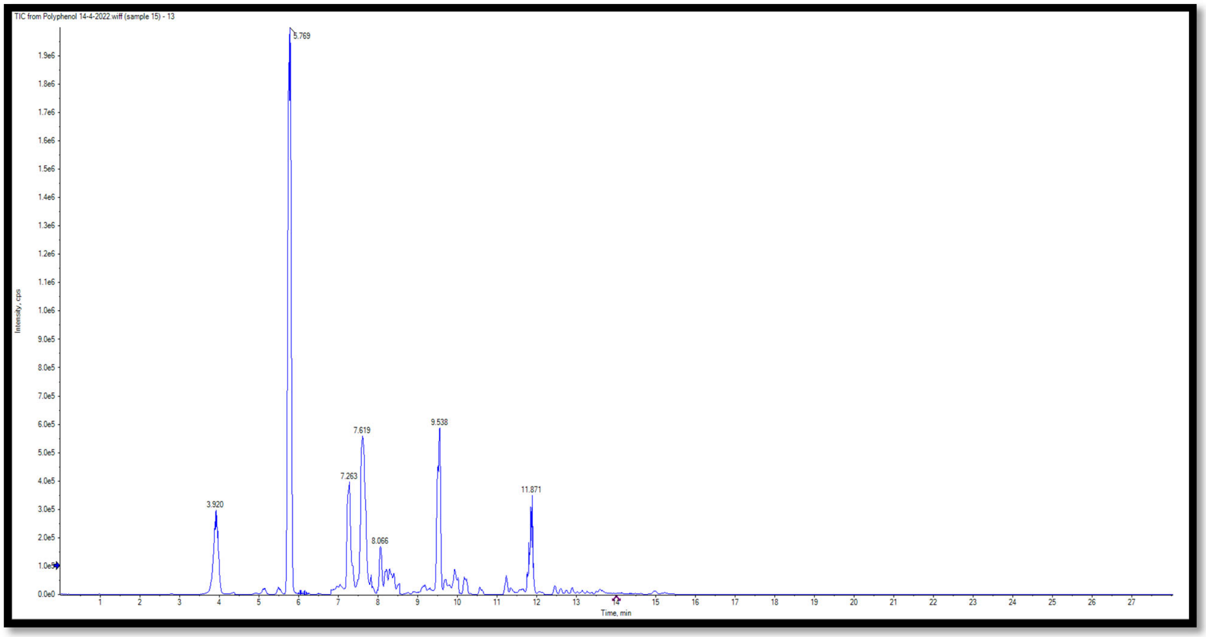

Figure S14. LC-MS/MS chromatogram obtained in MRM mode of 70% ethanolic extract of treatment no. 13 callus.

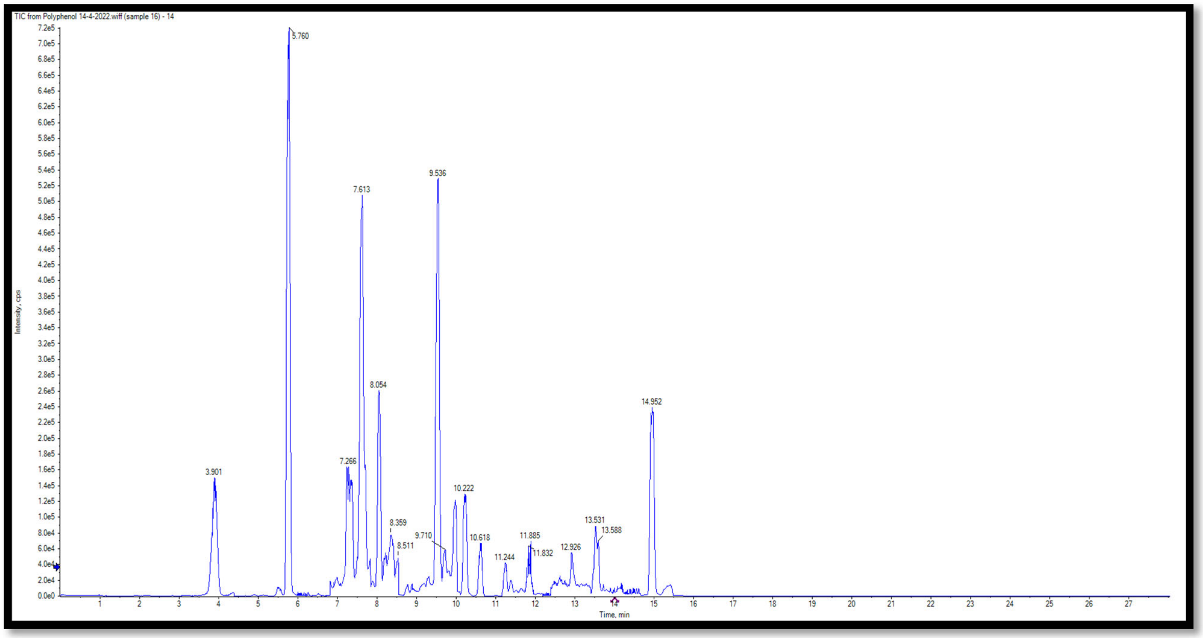

Figure S15. LC-MS/MS chromatogram obtained in MRM mode of 70% ethanolic extract of treatment no. 14 callus.

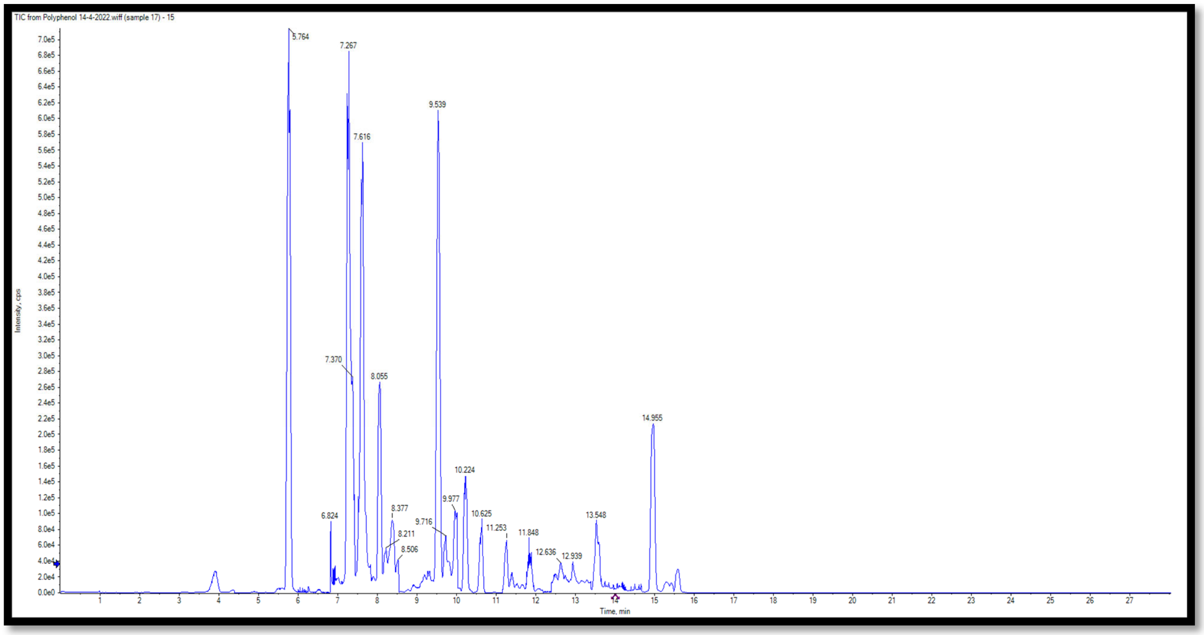

Figure S16. LC-MS/MS chromatogram obtained in MRM mode of 70% ethanolic extract of treatment no. 15 callus.

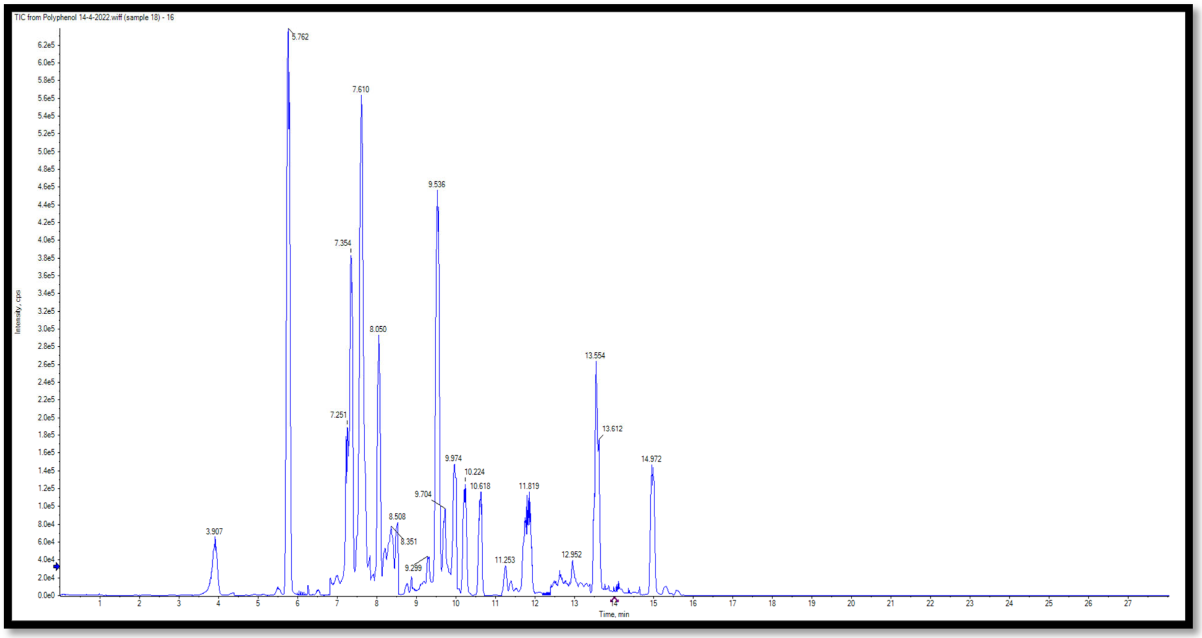

Figure S17. LC-MS/MS chromatogram obtained in MRM mode of 70% ethanolic extract of treatment no. 16 callus.

Table S1: The multiple reaction monitoring transitions and the optimized mass spectrometer parameters

Name	Q1 (m/z)	Q3 (m/z)	Expected RT (min)	CE (V)	CXP (V)	DP (V)
Gallic acid	168.9	124.9	3.9	-30	-11	-110
	168.9	79	3.9	-30	-11	-110
3.4-Dihydroxybenzoic acid	152.9	109	5.8	-40	-5	-75
	152.9	90.9	5.8	-20	-7	-75
Catechin	288.8	244.9	7.3	-16	-8	-40
	288.8	109	7.3	-32	-8	-40
Methyl gallate	183	124	7.5	-30	-10	-110
	183	140	7.5	-30	-10	-110
Chlorogenic acid	355.1	163	7.8	21	10	46
	355.1	89	7.8	75	14	46
Caffeic acid	178.9	135	8	-22	-9	-115
	178.9	107	8	-30	-7	-115
Syringic acid	196.9	122.8	8.4	-24	-5	-30
	196.9	181.9	8.4	-12	-5	-30
Coumaric acid	162.9	119	9.5	-20	-7	-90
	162.9	93	9.5	-40	-5	-90
Vanillin	151	136	9.6	-12	-9	-140
	151	92	9.6	-16	-7	-140
Rutin	609	299.9	9.7	-48	-15	-230
	609	270.9	9.7	-70	-9	-230
Ellagic acid	301	145	9.9	-40	-14	-120
	301	245	9.9	-38	-14	-120
Ferulic acid	192.8	133.9	10.2	-16	-5	-25
	192.8	177.9	10.2	-12	-5	-25
Myricetin	317	179	11.7	-19	-10	-100
	317	137	11.7	-26	-10	-100
Daidzein	255.1	199	13.4	28	10	125
	255.1	91.1	13.4	44	10	125
Luteolin	284.7	132.9	13.5	-38	-10	-50
	284.7	150.9	13.5	-26	-10	-50
Querectin	301	151	13.6	-28	-9	-50
	301	178.8	13.6	-20	-7	-50
Cinnamic acid	146.9	102.6	14.2	-17	-6	-60
	146.9	77	14.2	-33	-6	-60
Naringenin	271	151	15	-24	-25	-130
	271	119	15	-34	-11	-130
Apigenin	269	151	15	-15	-7	-35
	269	117	15	-15	-7	-35
Kaempferol	284.7	93	15.3	-46	-10	-120
	284.7	116.8	15.3	-52	-10	-120
Hesperetin	301	164	15.6	-23	-10	-125
	301	136	15.6	-38	-10	-125

Table S2: Different plant growth regulators used for *M. indica* callus induction.

Treatment No.	Media Composition
1.	Control (MS free treatment)
2.	MS + 0.25 gm CH + 1 mg/ L 2,4-D + 0.33 / L 2-iP
3.	MS + 0.25 gm CH + 1 mg/ L 2,4-D + 0.33 mg/ L BAP
4.	MS + 0.25 gm CH + 2 mg/ L 2,4-D
5.	MS + 0.25 gm CH + 2 mg/ L 2,4-D + 0.33 mg/ L 2-iP
6.	MS + 0.25 gm CH + 2 mg/ L 2,4-D + 0.33 mg/ L BAP
7.	MS + 0.25 gm CH + 4 mg/ L 2,4-D
8.	MS + 0.25 gm CH + 4 mg/ L 2,4-D + 0.33 mg/ L 2-iP
9.	MS + 0.25 gm CH + 4 mg/ L 2,4-D + 0.33 mg/ L BAP
10.	MS + 0.25 gm CH + 1 mg/ L Picloram
11.	MS + 0.25 gm CH + 1 mg/ L Picloram + 0.33 mg/ L 2-iP
12.	MS + 0.25 gm CH + 1 mg/ L Picloram + 0.33 mg/ L BAP
13.	MS + 0.25 gm CH + + 2 mg/ L Picloram
14.	MS + 0.25 gm CH + 2 mg/ L Picloram + 0.33 mg/ L 2-iP
15.	MS + 0.25 gm CH + 2 mg/ L Picloram + 0.33 mg/ L BAP
16.	MS + 0.25 gm CH + 4 mg/ L Picloram
17.	MS + 0.25 gm CH +4 mg/ L Picloram + 0.33 mg/ L 2-iP
18.	MS + 0.25 gm CH + 4 mg/ L Picloram + 0.33 mg/ L BAP

MS = Murashige and Skoog media   2, 4-D = 2, 4 Dichlorophenoxy acetic acid

BAP = Benzyl amino purine   2IP = 2-iso-pentenyl adenine

CH = Casein hydrolysate
